# Reliability and validity of the Japanese version of the Ocular pain assessment survey (OPAS-J)

**DOI:** 10.1038/s41598-023-36740-x

**Published:** 2023-06-23

**Authors:** Ryutaro Yamanishi, Natsume Suzuki, Miki Uchino, Motoko Kawashima, Kazuo Tsubota, Kazuno Negishi

**Affiliations:** 1 Department of Ophthalmology, Keio University School of Medicine, Tokyo, Japan; 2 Tsubota Laboratory, Inc., Tokyo, Japan

**Keywords:** Eye manifestations, Pain

## Abstract

This study aimed to determine the reliability and validity of the Japanese version of the Ocular Pain Assessment Survey (OPAS-J) to measure ocular pain and quality of life. A multi-institutional cross-sectional study was conducted on participants with and without ocular pain. The Wong–Baker FACES® Pain Rating Scale served as the gold standard for measuring the intensity of ocular pain. Sixty-four participants who visited two clinics located in Japan between May 2019 and October 2019 were included in the study. The OPAS was translated and culturally adapted to Japanese. The internal consistency of the OPAS-J was assessed using Cronbach’s alpha coefficient. Twenty-four (37.5%) and 40 (62.5%) participants were classified as having ocular pain and no ocular pain, respectively. All dimensions of the OPAS-J had good reliability, with a Cronbach’s alpha coefficient of 0.870 for ocular pain intensity over the past 24 h and 0.874, 0.899, 0.874, 0.871, and 0.876 for ocular pain intensity over the past 2 weeks, non-ocular pain, interference with quality of life, aggravating factors, and associated factors, respectively. The OPAS-J is a reliable and responsive tool that can be used to quantify ocular pain intensity.

## Introduction

The chief complaint of patients seeking ophthalmic medical help is ocular pain^1^. Neuropathic pain is defined as pain arising as a direct consequence of a lesion or disease affecting the somatosensory system, sustained by dysfunctional elements in the nociceptive system^2^, and can also occur in the cornea, which is the most richly innervated tissue in the body^3^.

Several studies attempted to evaluate patients with neuropathic ocular pain with dry eye-related questionnaires, such as the Ocular Surface Disease Index (OSDI)^4^, and Dry Eye related Quality of life Score (DEQS)^5^.

The Ocular Pain Assessment Survey (OPAS) is a validated questionnaire for ocular pain that is specifically designed to assess corneal and ocular surface pain and related quality of life (QoL) changes^6^. Currently, the reliability and validity of the OPAS have not yet been confirmed in Japan. Kim et al^7^ (Korea) and Yildiz-Tas et al^8^ (Turkey) have already assessed ocular pain using the OPAS.

Determining the reliability and validity of the Japanese version of the OPAS (OPAS-J) is essential for epidemiological and symptomatic comparisons with other countries.

## Results

Table 1 presents the characteristics of this study participants. All participants responded to the questionnaires, completed the examinations, and were eligible for the study. A total of 64 participants were included in this study. The average age was 63.9 ± 13.3 years, and 89.1% (n = 57) of the participants were women. Using the criteria of the gold standard Wong–Baker FACES® Pain Rating Scale, 40 and 24 patients were classified as presenting with ocular pain (62.5%) and presenting with no pain (37.5%), respectively. There were eight participants with meibomian gland dysfunction. No participants had allergic or atopic keratoconjunctivitis or after refractive surgery. Furthermore, there was no participants having history of Stevens-Johnson Syndrome and Toxic Epidermal Necrolysis, ocular pemphigoid or Graft Versus Host Disease, as well.

**Table 1:** Characteristics of study participants.

		Total	No pain	Pain	*p* value	Non-DED	DED	*p* value
		n = 64	n = 40	n = 24		n = 22	n = 42	
Age (year)		63.9 ± 13.3	65.1 ± 14.5	61.9 ± 11.1	0.37	63.5 ± 12.5	64.1 ± 13.9	0.86
Gender, woman (%)		57 (89.1)	36 (90.0)	21 (87.5)	0.76	17 (77.3)	40 (95.2)	0.03
Tear break up time (s)		5.0 ± 2.9	4.8 ± 2.6	5.3 ± 3.3	0.53	8.4 ± 1.5	3.2 ± 1.3	< .0001
Schirmer 1 score (mm)		3.8 ± 3.5	3.4 ± 2.8	5.2 ± 5.0	0.32	4.5 ± 3.5	3.4 ± 3.5	0.49
Conjunctival and corneal staining		1.3 ± 1.9	1.3 ± 2.1	1.2 ± 1.6	0.79	0.6 ± 1.1	1.6 ± 2.1	0.16
Dry eye related quality of life score		31.5 ± 22.6	26.3 ± 21.8	41.5 ± 21.1	0.01	28.2 ± 20.8	33.3 ± 23.6	0.41
Subscale of the OPAS-J	Ocular pain intensity (past 24 h)	1.5 ± 1.8	0.9 ± 1.4	2.4 ± 1.9	0.003	1.3 ± 1.9	1.6 ± 1.7	0.58
	Ocular pain intensity (past 2 weeks)	1.9 ± 2.3	1.3 ± 2.2	2.8 ± 2.2	0.02	1.3 ± 2.2	2.3 ± 2.3	0.12
	Non-ocular pain intensity	2.0 ± 2.8	1.2 ± 2.1	2.9 ± 3.3	0.03	1.6 ± 2.7	2.2 ± 2.9	0.46
	Interference with quality of life	1.9 ± 2.1	1.0 ± 1.3	3.1 ± 2.3	< .0001	1.8 ± 2.1	2.0 ± 2.1	0.71
	Aggravating factors	2.5 ± 2.8	1.7 ± 2.2	3.5 ± 3.2	0.02	1.7 ± 2.1	3.1 ± 3.1	
	Associated factors	2.2 ± 2.2	1.4 ± 1.4	3.2 ± 2.7	0.005	1.5 ± 1.7	2.7 ± 2.4	

*DED* Dry eye disease.

*OPAS-J* Japanese version of the Ocular Pain Assessment Survey.

Two-tailed t-tests were used for continuous variables and χ2 tests were used for categorical variables.

The mean values for tear break up time (TBUT), Schirmer I score, and conjunctival and corneal fluorescein staining were 5.0 ± 2.9 s, 3.8 ± 3.5 mm, and 1.3 ± 1.9, respectively. DEQS (range 0–100) was 31.5 ± 22.6.

The scores for each element of the OPAS-J were calculated according to the original version (0 = pain absent, > 0 = pain present). Ocular pain intensity (range 0–10) was 1.5 ± 1.8 (past 24 h) and 1.9 ± 2.3 (past 2 weeks). Non-ocular pain intensity (range 0–10) was 2.0 ± 2.8. Interference with QoL (range 0–10) was 1.9 ± 2.1. Aggravating and associated factors were 2.5 ± 2.8 and 2.2 ± 2.2, respectively.

A subanalysis classified by presence of pain was conducted. No significant differences were found in TBUT, Schirmer${\text{I}}$score, or conjunctival and corneal fluorescein staining between the pain and no-pain groups (all *p* > 0.05). In contrast, DEQS was significantly higher in the pain group than in the no pain group (41.5 ± 21.1 vs. 26.3 ± 21.8, respectively). All OPAS-J scores were significantly higher (worse) in the pain group (all *p* < 0.05).

The results, classified according to the presence or absence of dry eye disease (DED), are shown in Table 1. DED was defined according to the Asia Dry Eye Society criteria^9^. Among the 42 participants with DED, 14 had ocular pain (12 women), and among the 22 participants with non-DED, 10 had ocular pain (9 women) (*p* = 0.34). The DED group scored higher on all subscales of the OPAS-J, although there were no significant differences between the two groups.

The overall severity of pain on the day was as follows: no pain in 40 participants (62.5%), 1–2 out of 10 in 16 participants (25.0%), 3–4 out of 10 in 1 participant (1.6%), 5–6 out of 10 in 5 participants (7.8%), and 7–8 out of 10 in 2 participants (3.1%). None of the participants was scored as having the worst pain (9–10).

All dimensions of the OPAS-J had good reliability, with a Cronbach’s alpha greater than or equal to 0.87. Table 2 presents the results. For internal consistency, the Cronbach’s alpha coefficient was 0.870 for ocular pain intensity over the past 24 h and 0.874, 0.899, 0.874, 0.871, and 0.876 for ocular pain intensity over the past 2 weeks, non-ocular pain, interference with QoL, aggravating and associated factors, respectively.

**Table 2:** Reliability for each subscale the Japanese version of the ocular pain assessment survey.

Subscale of the OPAS-J	Number of questions	Cronbach's alpha		
		Total	No pain	Pain
Ocular pain intensity (past 24 h)	3	0.870	0.777	0.872
Ocular pain intensity (past 2 weeks)	3	0.874	0.752	0.877
Non-ocular pain intensity	3	0.899	0.938	0.894
Interference with quality of life	7	0.874	0.775	0.873
Aggravating factors	2	0.871	0.726	0.875
Associated factors	4	0.876	0.747	0.875

*OPAS-J* Japanese version of the ocular pain assessment survey.

**Figure 1:**
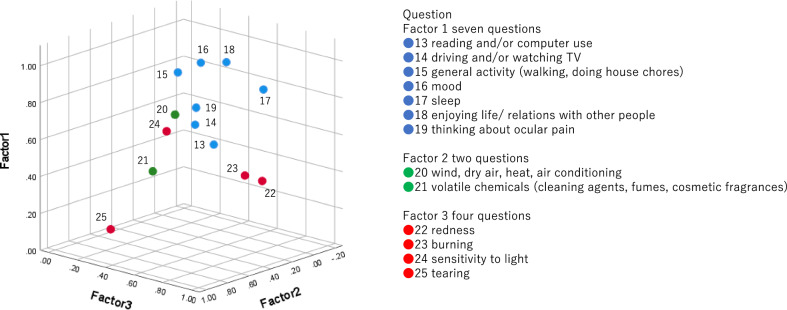
Three subscales of the Ocular Pain Assessment Survey (OPAS-J) as determined by factor analysis. These were in accordance with the subscales that are used in the original version of the OPAS: Factor 1 comprised questions assessing the interference with quality of life, Factor 2 comprised questions assessing aggravating factors, and Factor 3 comprised questions assessing associated factors.

Factor validity was assessed using confirmatory factor analysis to determine the subscales. As shown in Fig. 1, Factor 1 (Cronbach’s alpha = 0.92) comprised questions assessing the interference with QoL (seven questions), Factor 2 (Cronbach’s alpha = 0.75) comprised questions assessing aggravating factors (two questions), and Factor 3 (Cronbach’s alpha = 0.65) comprised questions assessing associated factors (four questions).

## Discussion

This multi-centered cross-sectional study assessed the reliability and validity of the Japanese version of the OPAS. The OPAS was specifically designed to evaluate the psychometric properties of ocular pain with numerical and quantifiable rating scales^6^. Further, it can be used for the evaluation of disease pain severity and its impact on related dimensions such as QoL, aggravating factors, and associated factors.

As shown in Table 2, all subscales of the OPAS-J showed high internal consistency. The factor analysis showed three subscales within the OPAS-J: QoL, aggravating factors, and associated factors, in accordance with the subscales in the original English version^6^.

Factor validity analysis showed that the associated factors had weak internal consistency among the four questions (Fig. 1). Tearing (Q25) showed a weak correlation, possibly due to the low prevalence of complaints of tearing. Twelve participants in the pain group and 18 in the no pain group reported no tearing, which may weaken the internal consistency of this question.

We performed the OPAS-J evaluation for participants with and without ocular pain. While there was a significant difference in each score on the OPAS-J when classified by the presence or absence of pain, the analysis classified by DED showed almost no significant difference in each score. The non-DED participants' mean value of Schirmer ${\text{I}}$ score in this study was shorter than that in the non-DED group of the Osaka study^10^ and the J-OSDI validation study^11^, indicating that tear function was likely not normal even though the participants were diagnosed as non-DED.

It suggests that neuropathic factors may have influenced the participants’ responses, although other factors such as epithelial damage to the cornea and conjunctiva, inflammation, the effects of friction, and asthenopia may also contribute. Kim et al. reported that the OPAS questionnaire can be a good option for evaluating whether patients have ocular neuropathic features and emphasized that burning sensation may be a key symptom^7^. In this study, burning sensation showed good consistency.

It is important to note that the predictive values of the OPAS-J in this study were specific to the prevalence of ocular pain at a given time point. The Neuropathic Pain Symptom Inventory for use in eye pain (NPSI-Eye) was suitably adapted for evaluating ocular pain, which showed good correlations between the NPSI-Eye and indicators of general ocular pain, whereas correlations between the NPSI-Eye and dry eye symptom severity and psychological health indices were lower^12^. The OPAS can be used to quantify pain intensity, measure the impact of pain on emotions and activities of daily living, and track symptom relief in patients with neuropathic ocular pain using a quantitative scoring system.

This study showed that there was a significant difference in the DEQS scores between participants with and without ocular pain. This seems to support the usefulness of the DEQS in assessing ocular pain, as shown in a previous report^5^. In addition, the DEQS is a questionnaire that can assess multidimensional QoL through questions regarding the impact on daily life^13^, suggesting that it may be similar to the questions on QoL in the OPAS-J. The DEQS is an evaluation scale mainly for DED patients, whereas the OPAS-J may be useful in a wider clinical setting as an evaluation scale for ocular pain in general. In this study, each element of the OPAS-J and Wong–Baker FACES® Pain Rating Scale showed strong correlation with DEQS (Supplementary Table 1).

This study was subject to the limitations of a small sample size, and unbalanced distribution of participants' age and sex, which may have diminished the external validity.

Firstly, although the number of participants reporting ocular pain was relatively small, suggesting that each item of the questionnaire may not have been adequately assessed, good reliability was indicated by Cronbach’s alpha in this study, and the internal validity of the present questionnaire was almost the same as that of the original OPAS.

Secondly, we assume that the reason for the unbalanced distribution of participants' ages was because the number of people who visit hospitals in Japan is skewed toward older adults. According to the Survey of Actual Medical Benefits in 2008, by the Japanese Ministry of Health, Labour and Welfare^14^, the hospital visit rate for those under 65 years is less than 0.2 visits/person, but the rate increases rapidly to 0.3 visits/person for those aged 65–69 years, 0.4 visits/person for those 70–74 years, and 0.6 visits/person for those 75–79 years. To examine differences by age group, a stratified analysis was conducted by dividing the group into those 65 years and older (n = 33) and those 64 years and younger (n = 31). In both groups, all dimensions of the OPAS-J had good reliability (Supplementary Table 2).

Thirdly, to the best of our knowledge, for the original version of the OPAS, there has been no discussion about the influence of participant sex. The report of Kim et al^7^ indicates that there were more women participants than men, but there was no discussion of any influence relating to sex. The report from Yildiz-Tas^8^ also did not discuss sex differences. According to the patient survey conducted by the Ministry of Health, Labour and Welfare of Japan on subjects who visit medical facilities, a higher percentage of women than men consult doctors^15^. As previous Japanese validation studies^11,13^ have shown the sex imbalance of participants, hospital-based surveys conducted in Japan may tend to bias the target population toward women. In addition, a stratified analysis among men and women was conducted, and the OPAS-J Cronbach’s alpha coefficients for men were higher than 0.70 for all dimensions, which is considered to be acceptable^16^ (Supplementary Table 2).

Fourthly, because this study was a multicenter study, a subtle bias may be present when conducting clinical examinations. Finally, as there was no continuity in the data obtained through questionnaires in this study, it is possible that the course of the study, including the treatment effects, has not been well investigated.

In conclusion, we demonstrated that the OPAS-J is a reliable and responsive tool that can be used to quantify ocular pain intensity.

## Methods

### Study population

This was a multi-institutional, cross-sectional study. Adult participants (aged over 20 years) of both sexes with and without complaints of ocular surface pain, who visited the Keio University Hospital (Tokyo, Japan), Fujishima Eye Clinic (Niigata, Japan) between May 2019 and October 2019 were included. Participants were consecutively enrolled from among those who visited the hospitals complaining of dry or irritated eyes during the study period and who agreed to participate in the study.

Written informed consent was obtained from all the participants. This study adhered to the tenets of the Declaration of Helsinki. This study was conducted as one of the Eye Pain Observational Study^5^, which the institutional review board of Keio University Hospital approved (approval number 20180027).

All participants underwent comprehensive ophthalmic evaluation for both eyes, including TBUT, Schirmer ${\text{I}}$ score, and conjunctival and corneal fluorescein staining^13^. Following the original version of OPAS^6^, the eye with the worse subjective symptoms was chosen for the analysis for each participant. Further, if both eyes had the same degree of subjective symptoms, the score of the right eye was included in the analysis.

The DEQS questionnaire was administered to the participants to assess the severity of dry eye-associated symptoms and the multifaceted effects of DED on daily life^13^. The score derived from this questionnaire is a subjective measure of DED symptoms, where 0 indicates the best score (no symptoms) and 100 indicates the worst score (maximum symptoms).

### OPAS-J questionnaire

The questions were divided into sections for analysis, used numerical rating scales to evaluate pain intensity of the worst eye (past 24 h and 2 weeks), non-ocular pain intensity, interference with QoL, aggravating factors, associated factors, and symptomatic relief. Each question was evaluated on a scale of 0–10 or 0–100 with increments of 1 or 10 units, respectively.

### Analysis

To compare general characteristics between ocular pain and no pain participants, two-tailed Student’s t-tests were used for continuous variables, whereas χ2 tests were used for categorical variables. Data are presented as the means ± standard deviations or proportions (%). The internal consistency of the OPAS-J was assessed using Cronbach’s alpha coefficient, with an alpha > 0.70 considered to be acceptable^16^. For factor validity, confirmatory factor analysis was conducted using promax rotation to determine whether the subscales in the OPAS-J clustered together in the same manner as in the original OPAS. All statistical analyses were performed using Statistical Analysis Software (SAS version 9.4, North Carolina, USA).

### Diagnostic analysis

Data were categorized according to the presence or absence of ocular pain using the gold standard Wong–Baker FACES® Pain Rating Scale (0 = pain absent, > 0 = pain present)^17^, which was adapted for the original version of the OPAS.

### Translation

To ensure a scientifically accurate translation and cross-cultural validation of the original version of the questionnaire, we used a forward–backward procedure to translate the OPAS from English to Japanese with reference to the Japanese translation of the OSDI^11^. Firstly, bilingual ophthalmologists independently performed forward translation and created a consensus version. Cultural adaptations were included to make the translated questionnaire easier for Japanese patients to understand. Secondly, this consensus version was translated into English by a native English-speaking researcher and evaluated for comprehension (Supplementary Fig. 1). Finally, the original translated and back-translated versions were carefully compared by a committee of experts to ensure equivalence of concepts.

## Supplementary Information

Supplementary Information 1.

Supplementary Information 2.

Supplementary Information 3.

## Data Availability

The datasets used and/or analyzed during the current study are available from the corresponding author on reasonable request.
