# Supplementary material for: Reliability and validity of the Japanese version of the Ocular pain assessment survey (OPAS-J)
Source: Sci Rep. 2023 Jun 23;13:10197. doi: 10.1038/s41598-023-36740-x (PMC10290131; doi:10.1038/s41598-023-36740-x)
Supplement: Supplementary file 1 — Supplementary Information 1. [file 41598_2023_36740_MOESM1_ESM.pdf]

Ocular Pain Assessment Survey (OPAS)

Please fill this form only if you have eye/ facial pain today or have filled this form before.

[Patient information sticker]

1. Please rate the overall severity of your pain today (0: no pain 10: More pain than have ever felt before)

Pain Scale

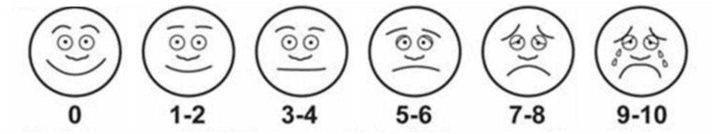

no pain slight pain moderate pain severe pain

2. On the diagram below, please shade the area where you have eye pain, and/or pain in the face and head region.

right eye **left eye**

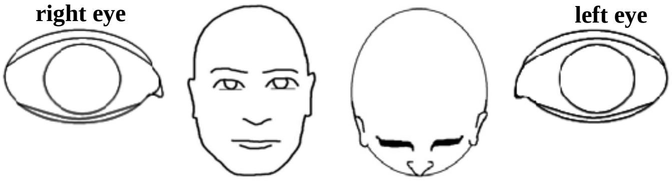

3. Do you have any long-standing pain elsewhere in your body ? □ No □ Yes
If "Yes", please indicate the area.

____

All question refer to pain in your worse eye   Please circle the level of your eye pain for the following:

eye pain intensity 24 hours

level of eye pain when it is most painful:

4. in the past 24 hours
no pain 0 1 2 3 4 5 6 7 8 9 10

level of eye pain when it is least painful:

5. in the past 24 hours
no pain 0 1 2 3 4 5 6 7 8 9 10

level of eye pain on average:

6. in the past 24 hours
no pain 0 1 2 3 4 5 6 7 8 9 10 severe pain

eye pain intensity 2 weeks

7. in the past 2 weeks
no pain 0 1 2 3 4 5 6 7 8 9 10 severe pain

8. in the past 2 weeks
no pain 0 1 2 3 4 5 6 7 8 9 10 severe pain

9. in the past 2 weeks
no pain 0 1 2 3 4 5 6 7 8 9 10 severe pain

non-eye pain

Circle the level of your worst non eye pain (pain at temple, back of head, cheek area):

10. in the past 24 hours
no pain 0 1 2 3 4 5 6 7 8 9 10

11. in the past 2 weeks
0 1 2 3 4 5 6 7 8 9 10 severe pain

Please circle the percentages of time you spend thinking about your non-eye pain (face, head):

12. not at all 0% 50% 100% all the time

quality of life

Please circle the number that best describes how much your pain has interfered with/ affected the following:

N/A

13. reading and (or) computer use

not at all 0 1 2 3 4 5 6 7 8 9 10 completely ☐

14. driving a car and/or watching TV

not at all 0 1 2 3 4 5 6 7 8 9 10 completely ☐

15. general activities (walking, housework)

not at all 0 1 2 3 4 5 6 7 8 9 10 completely ☐

16. mood

not at all 0 1 2 3 4 5 6 7 8 9 10 completely ☐

17. sleep

not at all 0 1 2 3 4 5 6 7 8 9 10 completely ☐

18. enjoying life/relationships with others

not at all 0 1 2 3 4 5 6 7 8 9 10 completely ☐

Please circle the percentage of time you spend thinking about your eye pain:

19. not at all 0% 50% 100% all the time

aggravating factors

Please circle how much your pain is increased when exposed to:

20. wind, dry air, heat, air conditioning

no change 0% 50% 100% severe increase

21. violate chemicals (cleaning agents, fumes)

no change 0% 50% 100% severe increase

associated factors

Please circle how often your eye pain is accompanied by the following symptoms:

22. redness

never 0% 50% 100% all the time

23. burning

never 0% 50% 100% all the time

24. sensitivity to light

never 0% 50% 100% all the time

25. tearing

never 0% 50% 100% all the time

symptom relief

Please circle how much pain relief you have experienced since the last visit:

N/A

26. eye pain

no relief 0% 10% 20% 30% 40% 50% 60% 70% 80% 90% 100% complete relief ☐

27. non-eye pain (face/head)

no relief 0% 10% 20% 30% 40% 50% 60% 70% 80% 90% 100% complete relief ☐

Name:______________________________ Date: ____________

Figure legend:

Supplementary Figure 1

The questionnaire was translated from the Japanese consensus version into English by a native English-speaking researcher. The actual OPAS-J questionnaire is only in Japanese. The questions were divided into sections for evaluating ocular pain intensity (Q4–6: in past 24h, Q7–9: in past 2 weeks), non-ocular pain intensity (Q10–12), interference with quality of life (Q13–19), aggravating factors (Q20,21), associated factors(Q22–25), and symptomatic relief (Q26,27).
