# Supplementary material for: Reliability and validity of the Japanese version of the Ocular pain assessment survey (OPAS-J)
Source: Sci Rep. 2023 Jun 23;13:10197. doi: 10.1038/s41598-023-36740-x (PMC10290131; doi:10.1038/s41598-023-36740-x)
Supplement: Supplementary file 2 — Supplementary Information 2. [file 41598_2023_36740_MOESM2_ESM.docx]

| Supplementary Table1 Correlation coefficients between Dry Eye related Quality of life Score and the Japanese version of the Ocular Pain Assessment Survey | | |
| --- | --- | --- |
|  | DEQS | |
|  | Rs* | p value |
| Ocular pain intensity (past 24h) | 0.53 | 0.06 |
| Ocular pain intensity (past 2 weeks) | 0.59 | 0.03 |
| Non-ocular pain intensity | 0.76 | 0.003 |
| Interference with quality of life: | 0.70 | 0.01 |
| Aggravating factors | 0.52 | 0.07 |
| Associated factors | 0.49 | 0.09 |
| Wong-Baker FACES® Pain Rating Scale | 0.34 | 0.007 |
| DEQS, Dry Eye related Quality of life Score | |  |
| * Spearman correlation score | |  |
