# Supplementary material for: Reliability and validity of the Japanese version of the Ocular pain assessment survey (OPAS-J)
Source: Sci Rep. 2023 Jun 23;13:10197. doi: 10.1038/s41598-023-36740-x (PMC10290131; doi:10.1038/s41598-023-36740-x)
Supplement: Supplementary file 3 — Supplementary Information 3. [file 41598_2023_36740_MOESM3_ESM.docx]

| Supplementary Table 2 Reliability for each subscale of the Japanese version of the Ocular Pain Assessment Survey stratified with sex and age | | | | | |
| --- | --- | --- | --- | --- | --- |
| Subscale of the OPAS-J | Number of questions | Cronbach's alpha | | | |
|  |  | Men | Women | Age under 65 years | Age over 65 years |
| Ocular pain intensity (past 24h) | 3 | 0.777 | 0.872 | 0.860 | 0.872 |
| Ocular pain intensity (past 2 weeks) | 3 | 0.752 | 0.877 | 0.880 | 0.877 |
| Non-ocular pain intensity | 3 | 0.938 | 0.894 | 0.868 | 0.894 |
| Interference with quality of life | 7 | 0.775 | 0.873 | 0.855 | 0.873 |
| Aggravating factors | 2 | 0.726 | 0.875 | 0.864 | 0.875 |
| Associated factors | 4 | 0.747 | 0.875 | 0.854 | 0.875 |
| OPAS-J, Japanese version of the Ocular Pain Assessment Survey | |  |  |  |  |
